# Zirconia nanocomposites with carbon and iron(iii) oxide for voltammetric detection of sub-nanomolar levels of methyl parathion†

**DOI:** 10.1039/c9na00589g

**Published:** 2019-10-29

**Authors:** Krishna Prasad Gannavarapu, V. Ganesh, Rajesh Babu Dandamudi

**Affiliations:** Department of Chemistry, Sri Sathya Sai Institute of Higher Learning Prasanthinilayam Campus, Puttaparthi Anantapur Dist. Andhra Pradesh 515134 India drajeshbabu@sssihl.edu.in +08555286919 +919441587413; Electrodics and Electrocatalysis Division, CSIR-Central Electrochemical Research Institute Karaikudi Tamil Nadu 630001 India

## Abstract

This study reports the synthesis of zirconia nanoparticles loaded on various carbon substrates, namely, reduced graphene oxide (Zr-r-GO), carbon nanotubes (Zr-CNT), and activated carbon (Zr-AC). In addition, a composite of zirconia–iron mixed oxide loaded on activated carbon (FeZr-AC) was also synthesized. The materials were characterized using SEM-EDX, HRTEM, FTIR, Raman spectroscopy, TGA and XRD. The FeZr-AC sample was found to have a nanorod like morphology. The samples were evaluated for their sensing potential towards methyl parathion (MP) using differential pulse voltammetry in a range of 0.0 V to −0.9 V (*vs.* Ag/AgCl) by drop casting on a glassy carbon electrode (GCE). All the modified GCEs best operated at a working potential of 0.4–0.9 V *vs.* Ag/AgCl/Cl^−^. FeZr-AC was found have the best limit of detection followed by Zr-AC, Zr-CNT and Zr-r-GO with their detection limits being 1.7 × 10^−9^ M, 17.2 ×10^−9^ M, 243.3 × 10^−9^ M and 534.0 × 10^−9^ M respectively. These materials were then used to detect MP in spiked sewage samples and showed good recoveries.

## Abstract 2

This study reports the synthesis of zirconia nanoparticles loaded on various carbon substrates, namely, reduced graphene oxide (Zr-r-GO), carbon nanotubes (Zr-CNT), and activated carbon (Zr-AC).

## Graphical abstract

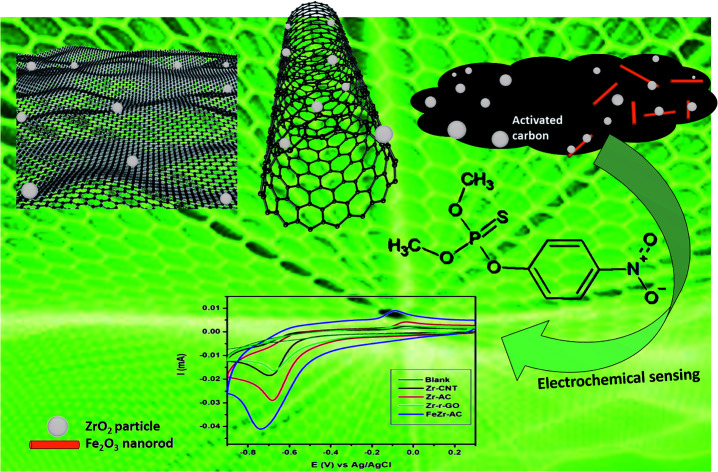

## Introduction

The use of excessive pesticides over the last few decades for better agricultural produce has resulted in the pollution of both ground and surface waters making innocent animals and humans unintentional targets.^1^ The accumulation of these pesticides leads to irreversible inhibition of acetylcholine esterase (AChE) causing increased concentration of acetylcholine at the receptor sites. This is implicated in conditions like muscular paralysis, bradycardia, both hyper and hypo tension, and convulsion and may even lead to death by asphyxiation.^2^ Thus, it becomes imperative that economic materials must be developed for rapid and effective ‘selective sensing’ of these toxic agents.^3^ Among OP pesticides, methyl parathion (MP) is widely used due to its good inhibition activity, easy availability and low cost.

A variety of analytical techniques like spectroscopy,^4–6^ gas and liquid chromatography,^7–9^ and gas chromatography coupled with mass spectrometry^10^ have been developed which are accurate and sensitive. However, they suffer from disadvantages of being expensive, tedious and time consuming with lengthy sample preparation. In this regard, disposable electrochemical sensors present an effective alternative being quick, selective, accurate and most importantly cheap.^11^ Many such sensors have been formulated to monitor and detect the levels of various OP pesticides. However, most of these methods essentially use enzyme-based techniques relying on the use of AChE and its inhibition activity to measure OP pesticides.^12,13^ These methods have shown acceptable low detection limits but suffer from the disadvantages of having higher costs and requiring careful handling.^12,14^

The presence of the nitro group in MP makes it electrochemically active and hence can be directly detected using electrochemical methods. There have been many reports, which make use of nanoparticles as part of the working electrode to directly detect MP. These include gold,^15,16^ silver, zinc oxide,^1,17^ zirconia nanoparticles^18–20^ and other metal oxides.^21,22^ Among all of these nanoparticles, the use of zirconia is of particular interest due to its affinity towards the phosphate group present in MP. These nanoparticles themselves have a large surface area and by virtue of their affinity towards the phosphate group, offer a high level of sensitivity and selectivity. There have been no reports on the sensitivity and development of zirconia loaded on CNTs and activated carbon and a single report exists on zirconia nanoparticle loaded graphene for OP sensing.^23^ The loading of the zirconia nanoparticles on different substrates is of interest because the association with different substrates might lead to better sensitivity of the working electrode due to varied conductivity and surface area of the substrates.

In the current work, we report a facile microwave mediated synthesis of zirconia nanoparticles loaded on graphene (Zr-r-GO), CNTs (Zr-CNT) and activated carbon (Zr-AC) derived from a spent mushroom substrate. In addition, we have also synthesized iron oxide–zirconia nanoparticles on AC (FeZr-AC) and studied their activity in the electrochemical sensing of MP. All the composites were characterized using SEM-EDX, HRTEM, FTIR, Raman spectroscopy, TGA and XRD. A lowest detection limit of 1.7 × 10^−9^ M was achieved for Fe–Zr-AC followed by Zr-AC, Zr-CNT and Zr-r-GO. All the composites sensed MP over a large range of linearity. The selectivity of the composites was retained even in the presence of interfering molecules and ions at high concentrations.

## Materials and methods

### Materials

The sawdust used for the preparation of the activated carbon was obtained from the mushroom culture facility in the department. Phosphoric acid, potassium hydroxide, ferrous sulphate, sodium hydroxide, sodium dihydrogen orthophosphate, disodium hydrogen orthophosphate, graphite, potassium hexacyanoferrate(iii) trihydrate, potassium hexacyanoferrate(ii), hydrogen peroxide, hydrazine hydrate, potassium hydroxide, nitric acid and sulphuric acid were of analytical grade and were obtained from Merck (http://www.merckmillipore.com). The MP standard was purchased from Sigma-Aldrich. All the solutions were prepared in Milli-Q water (resistivity = 18.2 MΩ cm at 25 °C).

### Instruments

The SEM and EDX data were obtained on a Jeol IT300 by putting a small amount of sample on carbon tape. The HRTEM images were obtained on a Jeol 2100 Plus instrument by drop casting the dispersed sample onto a copper grid. TGA was performed on a TA SDTQ600 by taking 5 mg of the sample in an alumina pan and heating until 800 °C at a rate of 10 °C min^−1^ under a nitrogen flow. Microwave reduction was carried out using a Mars 6 (CEM). Raman spectra were obtained using a Thermo Scientific DSR Raman microscope equipped with a 780 nm laser. FTIR spectra were recorded on an Agilent Cary 630 FTIR spectrometer in ATR mode. Powder XRD patterns were recorded on a Panalytical Xpert-3. GC-MS analysis was carried out on a Shimadzu QP 2010 SE equipped with an AOC 20i autosampler.

## Methods

### Synthesis of activated carbon

The sawdust precursor^24^ received from the mushroom cultivation facility of the department was washed with hot water, filtered and dried at 120 °C. The dried material was taken in a round bottom flask and charred with 60% *ortho*-phosphoric acid by heating it up to 150 °C. The obtained char was annealed at 500 °C in a tube furnace at a ramp rate of 5 °C min^−1^ under a flow of nitrogen at 100 mL min^−1^. Thus, the obtained product was washed with copious amounts of distilled water and ethanol until neutral pH, dried at 70 °C in a vacuum oven overnight and labelled SDAC500.

SDAC500 was mixed with a solution of 3 M KOH such that the SDAC500/KOH ratio is 1 : 3. The dry mixture was annealed at 800 °C in a tube furnace, at a ramp rate of 5 °C min under a flow of nitrogen at 100 mL min^−1^. Then, the obtained product was washed with copious amounts of distilled water and ethanol, dried at 70 °C in a vacuum oven overnight and labelled SDAC800.^25^

### Synthesis of graphene oxide (GO)

GO was prepared using the well-known Hummers process.^26^ In a typical experiment, 1 g of graphite powder along with 0.5 g of sodium nitrate was added to 23 mL of 98% sulphuric acid and cooled to 0 °C in an ice bath. To this mixture, 30 g of potassium permanganate was added with vigorous stirring ensuring that the temperature did not increase above 20 °C. This mixture was maintained at 35 °C for 30 min. The progress of the reaction was indicated by the formation of a brown paste with decrease in effervescence at the end of 20 min. At the end of 30 min, 46 mL of water was carefully added to the reaction mixture, which resulted in violent effervescence along with an increase in temperature to 98 °C. The reaction mixture was maintained at this temperature for 15 min. Later, the reaction mixture was further diluted with 140 mL of water and treated with hydrogen peroxide, which resulted in the mixture turning yellow. This was filtered while hot and washed with 70% ethanol and water for two cycles. The precipitate was then re-dispersed in 320 mL of water and dialyzed until the total dissolved salt content was constant in water. Finally, the residue was centrifuged at 10 000 rpm and the resultant precipitate was dried in a vacuum.

### Functionalization of SDAC800 and mwCNTs

mwCNTs (0.5 to 1.0 μm in length with their OD and ID being 70 nm and 20 nm respectively) and SDAC500 were added to concentrated HNO_3_ in a 10 : 1 wt/vol ratio and microwave irradiated with 1000 watts power in a Mars CEM EasyPrep vessel at 100 °C for 30 min. The reaction mixture was then cooled down to room temperature and filtered under vacuum using a 0.22 μm PTFE membrane filter. The product was then washed thoroughly with water (Milli-Q) and dried in a vacuum oven overnight. The products were collected and labeled f-mwCNT and f-SDAC800.

### Synthesis of ZrO_2_ nanoparticle loaded r-GO, CNT and SDAC800

#### ZrO_2_ loaded r-GO

100 mg of the synthesized GO above was dispersed in 50 mL of Milli-Q water by ultra-sonication to which 50 mL of 10 mM ZrOCl_2_ solution was added and sonicated for 20 min. To this well-dispersed solution, 1 mL of hydrazine hydrate was added and the mixture was microwave irradiated with 1000 watts power in a Mars CEM EasyPrep vessel at 150 °C for 40 min. The obtained product was filtered under vacuum using 0.22 μm PTFE membrane filters, washed thoroughly with water (Milli-Q) and dried in a vacuum overnight. The product was weighed and labelled Zr-r-GO.

#### ZrO_2_ loaded mwCNT/SDAC800

f-mwCNTs (300 mg) and f-SDAC800 (300 mg) were dispersed in 100 mL of 10 mM ZrOCl_2_ solution and sonicated for 20 min. This well-dispersed solution was transferred to a Mars CEM EasyPrep vessel and microwave irradiated with 1000 watts power at 150 °C for 40 min. The obtained product was filtered under vacuum using 0.22 μm PTFE membrane filters, washed thoroughly with water (Milli-Q) and dried in a vacuum overnight. The product was weighed and labelled Zr-CNT and Zr-AC respectively.

#### FeO_*x*_-ZrO_2_ nanorods loaded on SDAC800

500 mg of SDAC800 was well dispersed in 200 mL of nitrogen purged Milli-Q water by ultra-sonication. To this solution, 700 mg of FeSO_4_·7H_2_O and 1.5 mL of 0.25 mM ZrOCl_2_ solution were added and heated to 60 °C in a microwave reactor initially. The pH of this solution was adjusted to 11 using 3 M NaOH solution and the resulting solution was transferred to a Mars CEM EasyPrep vessel and microwave irradiated with 1000 watts power at 150 °C for 20 min. The obtained product was filtered under vacuum using 0.22 μm PTFE membrane filters, washed thoroughly with water (Milli-Q) and dried in a vacuum overnight. The product was weighed and labeled FeZr-AC.

### Electrochemical measurements

Electrochemical studies involving cyclic voltammetry and differential pulse voltammetry were carried out in a three electrode system setup on a Biologic VSP300, a potentiostat/galvanostat system. The working electrode used was a glassy carbon electrode (GCE) which was polished using 0.3 and 0.05 μm alumina slurry and was further cleaned by sonicating in water and ethanol. The counter electrode used was a platinum wire cleaned using dilute HCL and the reference electrode used was a Ag/AgCl/Cl^−^ electrode. The main working electrode for the sensing studies was fabricated by drop casting 10 μl of the slurry of the electrode materials pre-dispersed (1 mg ml^−1^) in Milli-Q water and by drying the same in a vacuum.

## Results and discussion

### Characterization

**Fig. 1:**
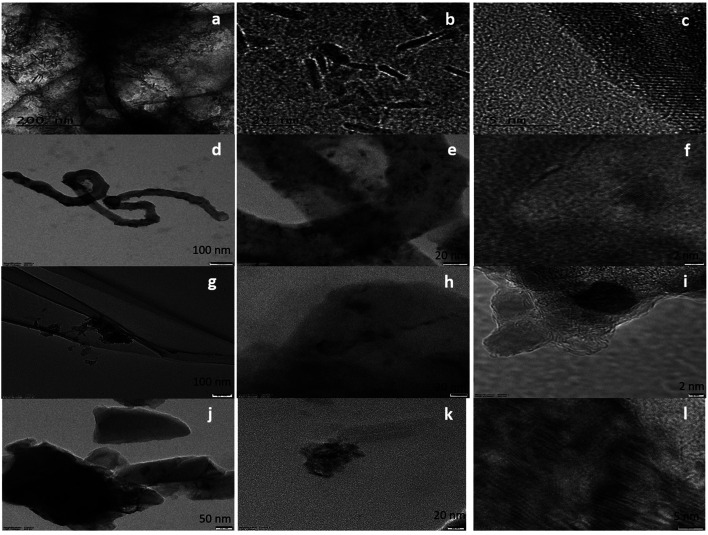
HRTEM images of (a–c) FeZr-AC, (d–f) ZrO_2_-CNT, (g–i) ZrO_2_-r-GO and (j–l) ZrO_2_-AC at different scales.

**Fig. 2:**
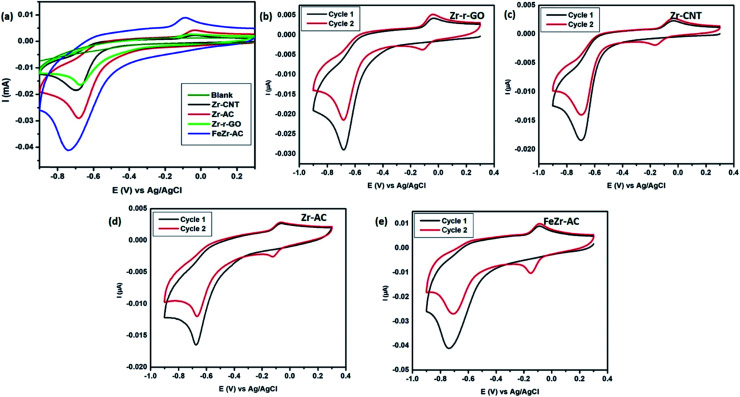
Cyclic voltammograms of the different materials *viz.*, Zr-r-GO, Zr-CNT, Zr-AC and FeZr-AC 2.5 μg ml^−1^ of MP (15 mM PB, pH 7.2) at a scan rate of 50 mV s^−1^. (a) Comparison of the materials; (b)–(e) cyclic voltammograms of the materials for cycle 1 and 2 to understand the mechanism. The CVs were recorded by sweeping from positive potential to negative potential from 0.3 V to −0.9 V.

The surface morphology of the samples was evaluated using SEM (Fig. S1†) and HRTEM. The HRTEM images showed the presence of ZrO_2_ nanoparticles (Fig. 1) in all samples. In the case of FeZr-AC, the particles can be seen in the form of nanorods (Fig. 2a and b). The EDX spectra of the samples were recorded to estimate the percentage loading of zirconia on various substrates. The results are summarized in Table 1. The EDX data indicated the presence of ZrO_2_ in the samples. Moreover, to confirm the homogeneous presence of the nanoparticles in all samples, XPS mapping was carried out at 184 eV for Zr 3d_3/2_, 287 eV for C 1s, 530 eV for O 1s and 730 eV for Fe 2p_1/2_ (Fig. S2†). The images clearly indicated the uniform presence of Zr in the composites Zr-r-Go, Zr-CNT and Zr-AC along with Fe in FeZr-AC.

**Table 1:** Loading of Zr on different substrates as obtained from EDX spectra

Sample	Loading (atomic %)
ZrO_2_-r-GO	3.84
ZrO_2_-CNT	3.19
ZrO_2_/AC	3.24
FeZr-AC (Fe, Zr)	3.89 (Fe), 2.22 (Zr)

The FTIR spectra showed the presence of the –COOH functionality in samples SDAC800 and mwCNT (Fig. S4†) as indicated by the increase in the intensities of *ν*_–OH_ at 3400 cm^−1^ and *ν*_–C=O_ at around 1610 cm^−1^ from SDAC800 and mwCNT to f-SDAC800 and f-mwCNT respectively. The FTIR spectra (Fig. S3a–c†) also indicated the formation of zirconia particles on various substrates. The small absorption peak at 470 cm^−1^ along with the peaks at 613 cm^−1^ and 819 cm^−1^ for Zr–O–Zr vibrations is indicative of ZrO_2_ particle formation.^27^ Zr-AC, Zr-CNT and Zr-r-GO have strong absorption bands at around 1700 cm^−1^ indicating the presence of *ν*_–C=O_, a reminiscent vibration due to functionalization to anchor the ZrO_2_ particles. However, this peak is absent in the FeZr-AC material as the activated carbon substrate here was not functionalized. The sharp peak at 3000 cm^−1^ in Zr-r-GO is due to the symmetric stretching of unsaturated –C–H bonds. The common peaks for all the samples were at 3500 cm^−1^, 1550 cm^−1^ and 1100 cm^−1^ and can be attributed to the *ν*_–OH_ symmetric stretching, skeletal C=C stretching and C–O stretching respectively.

The Raman spectra (Fig. S3d–g†) of the samples indicated the graphitic nature of the substrates (Fig. S5†). The peaks at 150 cm^−1^, 300 cm^−1^, 450 cm^−1^, and 650 cm^−1^ correspond to zirconia (shown in the insets) in its tetragonal form.^28,29^ The presence of these peaks clearly states the formation of ZrO_2_ nanoparticles. In the case of FeZr-AC, the peaks at 255 cm^−1^ (shoulder), 299 cm^−1^ and 613 cm^−1^ correspond to α-Fe_2_O_3_. These peaks originate from E_g_ modes of α-Fe_2_O_3_. The decrease in the intensity of the G-band along with the increase of the same in the D-band when compared to the substrates clearly indicates the presence of zirconia nanoparticles on the substrates (Table S1†).

The XRD spectra (Fig. S6†) also support the conclusions drawn from the other techniques. The 2*θ* peaks at 30.20, 35.28 and 50.76 correspond to the (101), (110) and (200) planes of ZrO_2_ in the tetragonal phase concurring with the Raman spectra. The FeZr-AC sample has shown 2*θ* peaks at 24.12, 33.11 and 63.96 which correspond to the (012), (104) and (300) planes of α-Fe_2_O_3_, strengthening the findings from Raman spectra. The peaks observed at 2*θ* values of 26.21 and 43.56 corresponding to the (002) and (100) lattice planes of carbon were also seen.

### Electrochemical studies

Cyclic voltammetry was employed in order to study the electrochemical response of different electrode materials towards MP. The cyclic voltammograms are shown in Fig. 2a. The reduction of MP occurs at −0.67 V, −0.69 V, −0.68 V and −0.73 V for Zr-r-GO, Zr-CNT, Zr-AC and FeZr-AC loaded GCE, respectively, while no reduction current for MP was observed with the plain GCE. It is also interesting to note that for the same amount of MP, the reduction peak current was maximum for FeZr-AC followed by Zr-AC, Zr-CNT and Zr-r-GO respectively. This may be attributed to the higher surface area of the materials in FeZr-AC and Zr-AC. Moreover, the improved performance of FeZr-AC may be due to improved conductance because of synergistic effects between Fe and Zr in the composite. The increased surface area also helps in better adsorption of MP leading to higher reduction currents.

### Electroanalytical studies

**Fig. 3:**
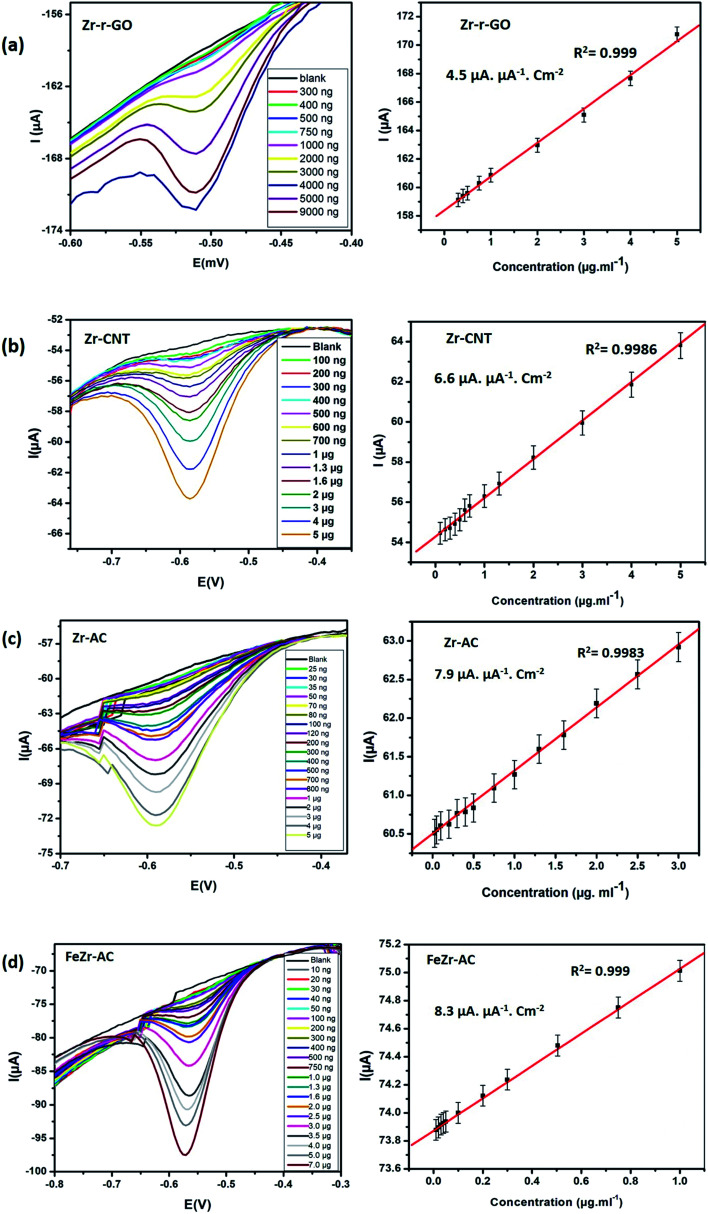
DPV voltammograms and the corresponding linearity plots obtained at increasing concentrations for (a) Zr-r-GO, (b) Zr-CNT, (c) Zr-AC and (d) FeZr-AC.

Differential pulse voltammetry (DPV) experiments were designed and carried out to understand and develop these materials as possible non-enzymatic analytical tools for the detection of MP. All the DPV experiments were carried out by sweeping the potential from 0 V to −0.9 V in 15 mM PB with pH 7.2 as the supporting electrolyte. The pulse height and pulse width for the experiment were standardized to be 50 mV with a step height of −5.0 V. The differential pulse voltammograms (Fig. 3) indicate that for all the studied materials, the reduction current increases systematically with increase in the concentration of MP. A minor shift in the reduction peak potential was observed to the negative side and this is explained by the possible increase in the resistance of the solution with increase in the concentration of MP. This shift is generally between 10 and 20 mV and the same has been noticed for all the materials. From Fig. 3, it is clear that all the materials maintain a linear range of detection across low to higher concentrations and the data for the same are summarized in Table 2.

**Table 2:** Limit of detection and linearity range of samples Zr-r-GO, Zr-CNT, Zr-AC and FeZr-AC and comparison with other electrode materials from the literature

Electrode material/type of electrode	Limit of detection (mol)	Linear concentration-range (mol)	Reference
Zr-r-GO/GCE	534.0 × 10^−9^	1.0 × 10^−6^ to 30.9 × 10^−6^	This work
Zr-CNT/GCE	243.3 × 10^−9^	0.3 × 10^−6^ to 34.3 × 10^−6^	This work
Zr-AC/GCE	17.2 × 10^−9^	85.9 × 10^−9^ to 68.7 × 10^−6^	This work
FeZr-AC/GCE	1.7 × 10^−9^	34.4 × 10^−9^ to 68.7 × 10^−6^	This work
ZrO_2_–Au	10.3 × 10^−9^	17.2 × 10^−9^ to 0.3 × 10^−6^	30
CNT/carbon paper	10.3 × 10^−9^	34.3 × 10^−9^ to 3.4 × 10^−6^	31
ZrO_2_/CPE	7.6 × 10^−9^	1.9 × 10^−8^ to 1.1 × 10^−5^	14
Au/MWCNTs	190 × 10^−9^	1.9 × 10^−6^ to 6.1 × 10^−5^	32
Mesoporous carbon/GCE	7.6 × 10^−9^	90 × 10^−9^ to 61 × 10^−6^	33
Silicate/GCE	10 × 10^−9^	1.0 × 10^−7^ to 1.0 × 10^−4^	34
GR-CS/GCE	2.7 × 10^−9^	13.7 × 10^−9^ to 1.37 × 10^−6^	35

Of the studied materials Zr-Ac and FeZr-AC showed particularly lower detection limits and exhibited linearity over a wider range of concentrations. The better activity of the materials derived from activated carbon can be attributed to their higher surface area and possible better conductivity. The better performance of FeZr-AC is attributed to the synergistic effect between Fe and Zr. To study this, we have recorded and compared the cyclic voltammograms of Zr-AC, FeZr-AC and Fe-AC (Fig. S9†). We observed that the material Fe-AC showed capacitive behavior when compared to FeZr-AC and Zr-AC. This is expected as iron is in the oxide form. We also found that it showed a weak signal for the analyte, MP, and so may not be a suitable sensor material.

### Interference studies

**Fig. 4:**
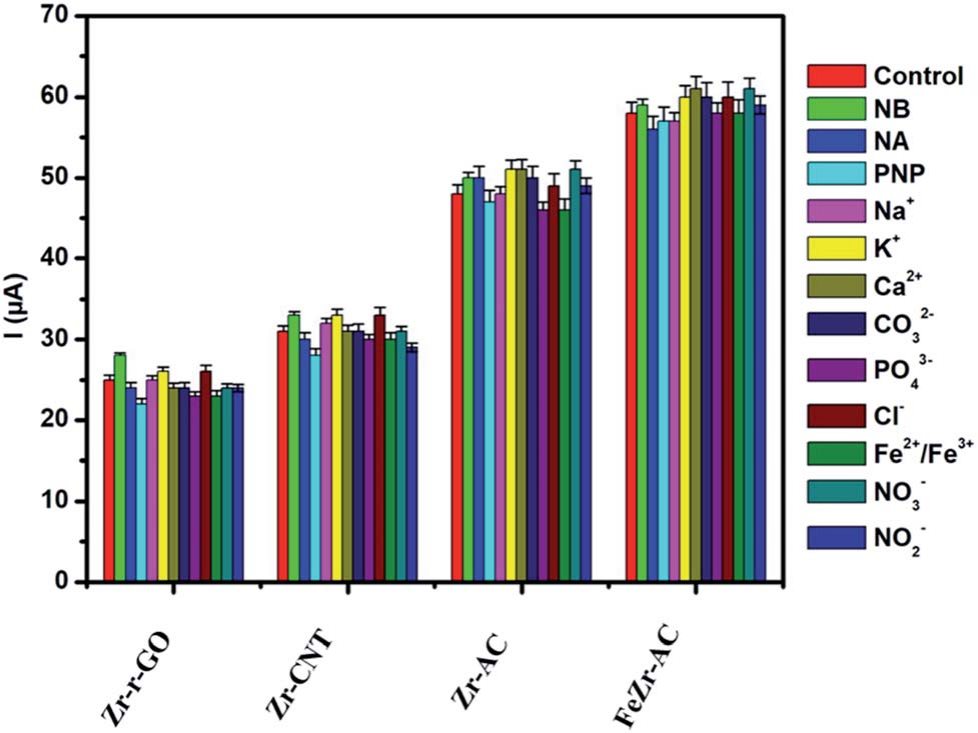
Interference studies performed on the materials Zr-r-GO, Zr-CNT, Zr-AC and FeZr-AC in 2.5 μg ml^−1^ of MP (15 mM PB, pH 7.2).

Selectivity plays a major role in determining the worth of any electrode material as a viable sensor. In order to ascertain the selectivity of the electrode materials towards MP without any ubiquity, DPV experiments were carried out in the presence of some of the commonly studied interfering ions and nitro aromatic compounds. The analysis was carried out in the presence of nitrobenzene (NB), *p*-nitrophenol (PNP) and *p*-nitroaniline (NA) in ten times higher concentration for each when compared to MP. A thousand-fold higher quantities of Na^+^, K^+^, Ca^2+^, CO_3_^2−^, PO_4_^2−^, Cl^−^, Fe^2+^/Fe^3+^, NO_3_^−^ and NO_2_^−^ were used for interference studies. The amount of MP was kept constant at 2.5 μg ml^−1^ and the results are summarized in Fig. 4. It was noticed that the interference current was less than 5% in the case of FeZr-AC and the highest of 8% in the case of Zr-r-GO, making a strong statement in favor of the high selectivity of the materials to MP. The higher interference in the case of Zr-r-GO can be expected due to possible π–π interactions with the aromatic compounds, which is not possible in the case of the AC substrate.

### Mechanism of sensing of MP

It is important to understand the mechanism of sensing MP in order to fabricate better sensors. An attempt has been made to elucidate the mechanism of electrochemical signal generation by MP with the help of cyclic voltammograms obtained (Fig. 2b–e). This has been thoroughly discussed in the ESI.†^18,36^ The CVs recorded without MP yielded no reduction peaks, asserting that there is no redox reaction happening. In addition, the reduction process occurring in the first step was studied at different scan rates of 10, 20, 30, 40, 60, 80 and 100 mV s^−1^ (Fig. S9†) to determine whether the process is diffusion controlled or surface confined. It was noticed that there was a steady increase in the reduction current with an increase in the scan rate and the slopes of the logarithmic plots of the corresponding peak current *vs.* scan rate were calculated to be 0.64, 0.71, 0.68 and 0.77 for Zr-r-GO, Zr-CNT, Zr-AC and FeZr-AC respectively. It is known that if this slope equals to 0.5 then the corresponding redox process is only diffusion controlled and if the value equals 1.0 then the process is a surface confined process.^37^ The slope values indicate that the process is both surface confined and diffusion controlled.

The presence of the zirconia nanoparticles primarily has led to the selectivity and sensitivity of the studied materials. It has been previously studied and understood that the interaction of zirconia with phosphate occurs *via* the inner sphere mechanism with the Zr–O bond interacting with P. This is however absent in the case of other moieties like SO_4_^2−^, NO^3−^ and Cl^−^.^38^ MP has a thiophosphoryl group which interacts with the zirconia present in the electrode materials. This strong interaction is deemed responsible for endowing the composites with good sensitivity and selectivity. This hypothesis is further strengthened due to weaker signals obtained from analytes like *p*-nitrophenol (PNP), nitrobenzene (NB), and *p*-nitroaniline (NA) which lack any phosphorus moiety.

Moreover, it has been observed that the FeZr-AC composites exhibit better sensitivity when compared to Zr-AC, Zr-CNT and Zr-r-GO composites. To further investigate this, the electrochemically active surface area (A) of these materials was calculated using the Randles–Sevcik equation and the conductance of the samples were calculated using the Nicholson equation by evaluating the electron rate transfer coefficient (*K*^o^_obs_) for the simple ferri–ferro redox system. The data are summarized in Table S2.† The results point out that the higher active surface area and better conductance of FeZr-AC help in achieving better sensitivity for the material when compared to others.

### Real sample analysis

In order to demonstrate the feasibility of the samples to be used as MP sensors in real samples, sewage water from the university site (SSSIHL, Puttaparthi) was collected and the analysis was carried out. Initially the water sample was filtered through a 0.22 μ membrane filter to remove the particulates and was analyzed for the presence of MP. The analysis of this sample showed no response for MP indicating the absence of the analyte in the water. Recovery studies were carried out by spiking this water sample with known concentrations of MP. Two different concentrations were used for each electrode material and the results are summarized in Table 3. All the samples showed good recovery values and demonstrate the potential usage of these as electrode materials for detecting MP in environmental samples. The recovery rates obtained by the electrochemical method have been verified using the standard GC-MS technique and the results are presented in Table S3 of the ESI.†

**Table 3:** Recovery studies for spiked sewage water along with the standard deviation where *n* = 3

	Zr-r-GO		Zr-CNT		Zr-AC		FeZr-AC	
Added MP (nM)	1220	1230	440	450	95	100	45	50
Found MP (nM)	1221.2 (±1.3)	1238.4 (±0.6)	442.3 (±2.3)	444.9 (±2.5)	96.5 (±2.6)	102.6 (±2.3)	46.5 (±1.4)	48.6 (±1.7)
Recovery (%)	100.1	100.7	100.5	98.8	101.5	102.6	103.3	97.2

In real samples, parathions are known to slowly oxidize to paraoxons. The presence of these should also be detected if assessment with respect to toxic effects of MP needs to be carried out in total.

## Conclusions

In the current work, we have demonstrated novel carbon based composites for selective and sensitive enzyme free electrochemical sensing for the detection of organo-phosphor class pesticide methyl parathions and have compared them for their sensitivity towards MP. A facile microwave mediated synthetic procedure was employed to synthesize zirconia loaded carbon substrates Zr-r-GO, Zr-CNT, Zr-AC and FeZr-AC which were characterized using SEM-EDX, HRTEM, FTIR, Raman spectroscopy, TGA and XRD. The FeZr-AC sample was found to have nanorod like morphology with the Zr loading being uniform in all the samples. The samples were evaluated for their sensing potential towards MP where FeZr-AC was found to be the most highly sensitive material followed by Zr-AC, Zr-CNT and Zr-r-GO respectively. It was also found that all the materials were highly selective towards MP even in the presence of other interfering molecules and ions, showing a high recovery percentage when spiked real samples were analyzed. We hope that these composite materials find application as disposable electrodes in MP detection. Further studies need to be conducted to detect MP in the presence of similar organophosphate pesticides.

## Conflicts of interest

There are no conflicts to declare.

## Supplementary Material

NA-001-C9NA00589G-s001
