# Supplementary material for: Zirconia nanocomposites with carbon and iron(iii) oxide for voltammetric detection of sub-nanomolar levels of methyl parathion
Source: Nanoscale Adv. 2019 Oct 29;1(12):4947–54. doi: 10.1039/c9na00589g (PMC9419288; doi:10.1039/c9na00589g)
Supplement: NA-001-C9NA00589G-s001 [file NA-001-C9NA00589G-s001.pdf]

Electronic Supplementary Material (ESI) for Nanoscale Advances.
This journal is © The Royal Society of Chemistry 2019

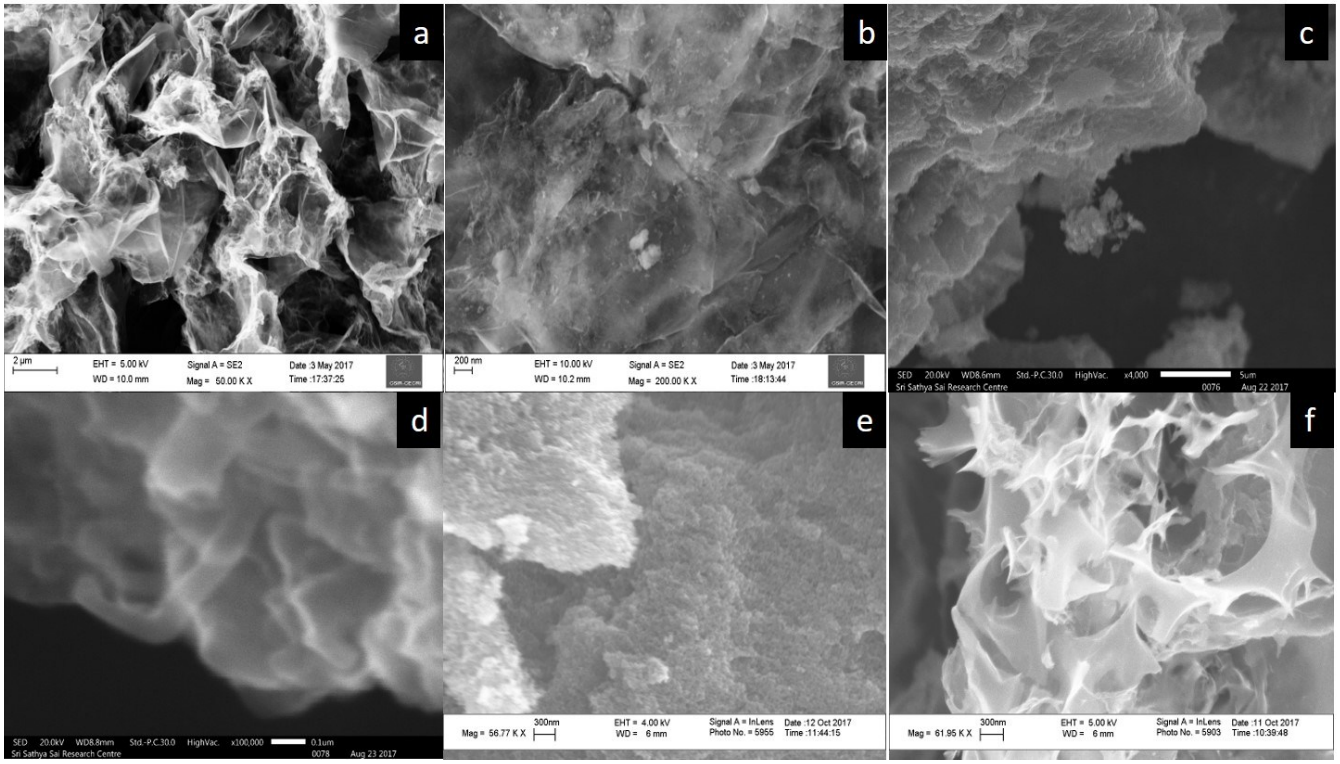

Figure S1: SEM images of (a) plain r-GO, (b) Zr-r-GO, (c) MWCNT, (d) f-MWCNT, (e) SDAC-500 and (f) SDAC-800.

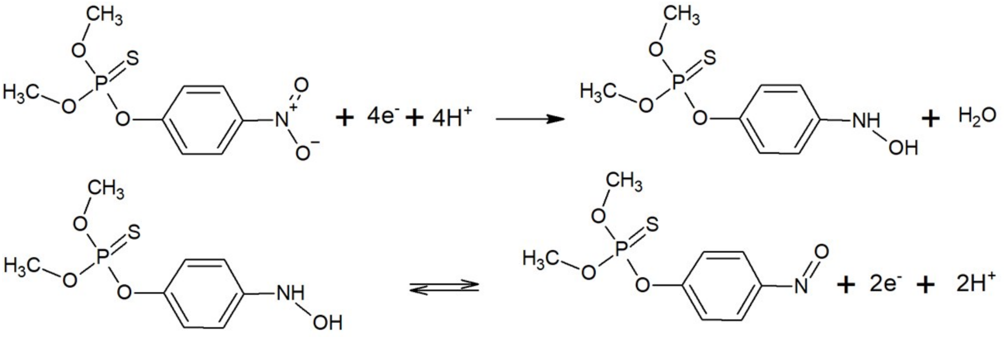

(1)

(2)

Scheme S1: Mechanism of electrochemical detection of MP

The CVs were recorded for two continuous cycles. In the first cycle, an intense and irreversible reduction peak was observed at around -0.6V and that can be attributed to the reduction of nitro group in MP converting it to hydroxylamine group (eq 1 in scheme S1). During the anodic sweep, the hydroxylamine obtained as a reduction product is oxidized at 0.03V generating a nitrosophenyl

group, which is reversibly reduced back to hydroxylamine (eq 2 in scheme S1) at -0.1 V in the next cathodic sweep step.

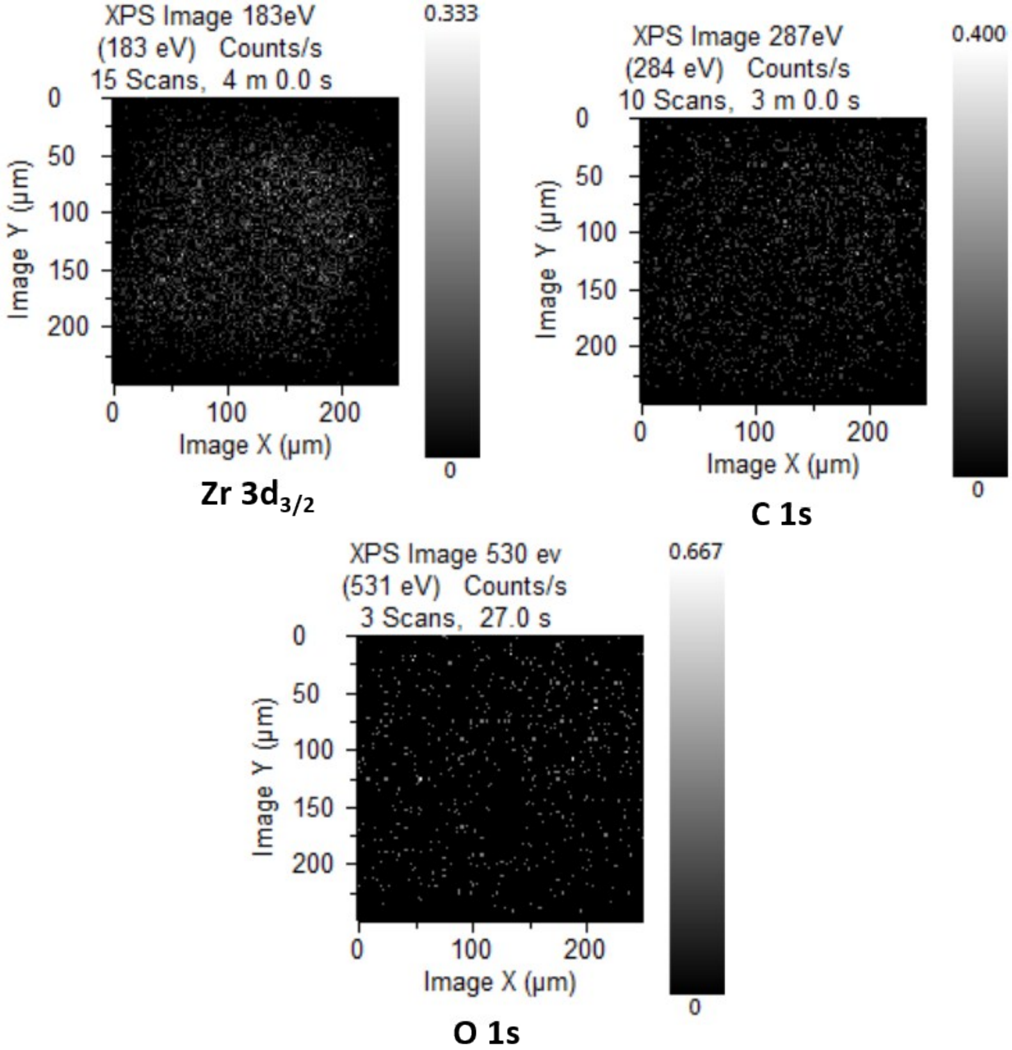

Figure S2 (a): XPS mapping images of Zr-r-GO.

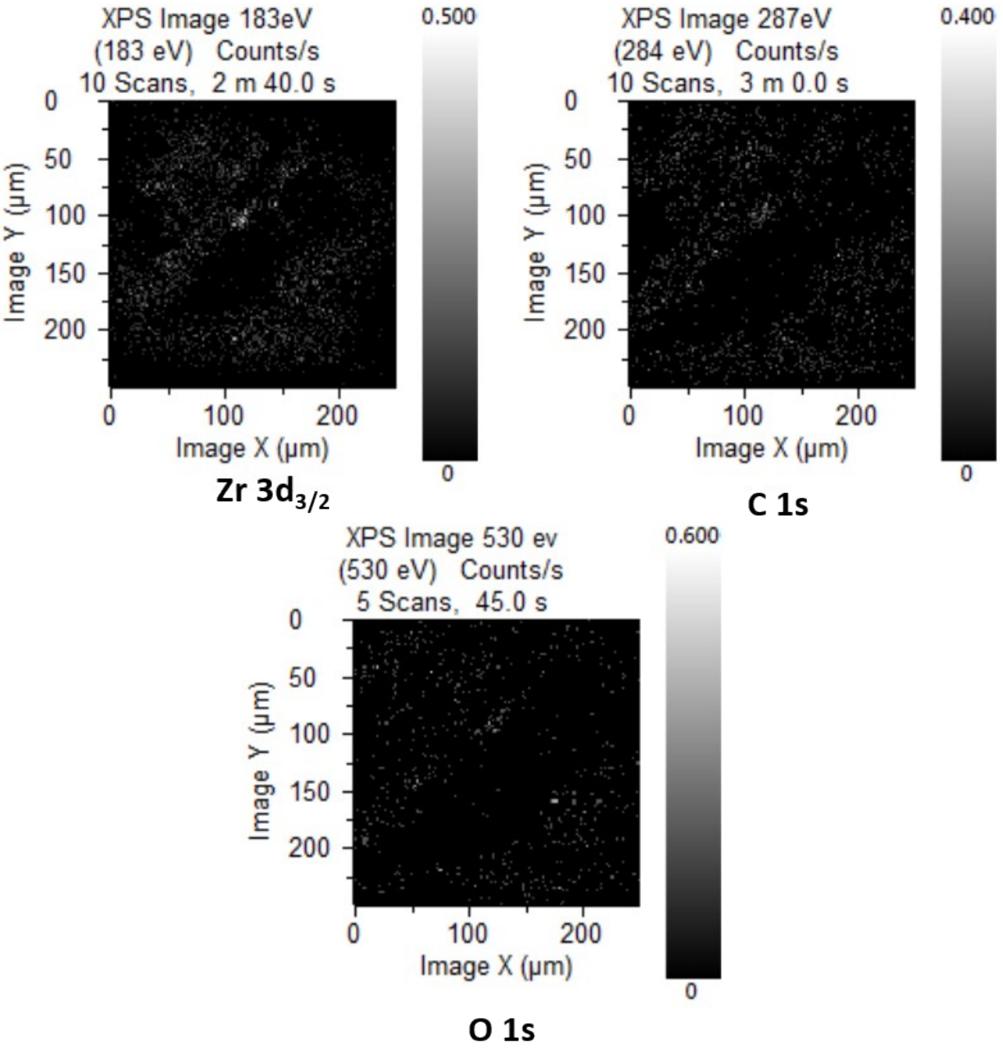

Figure S2 (b): XPS mapping images of Zr-CNT.

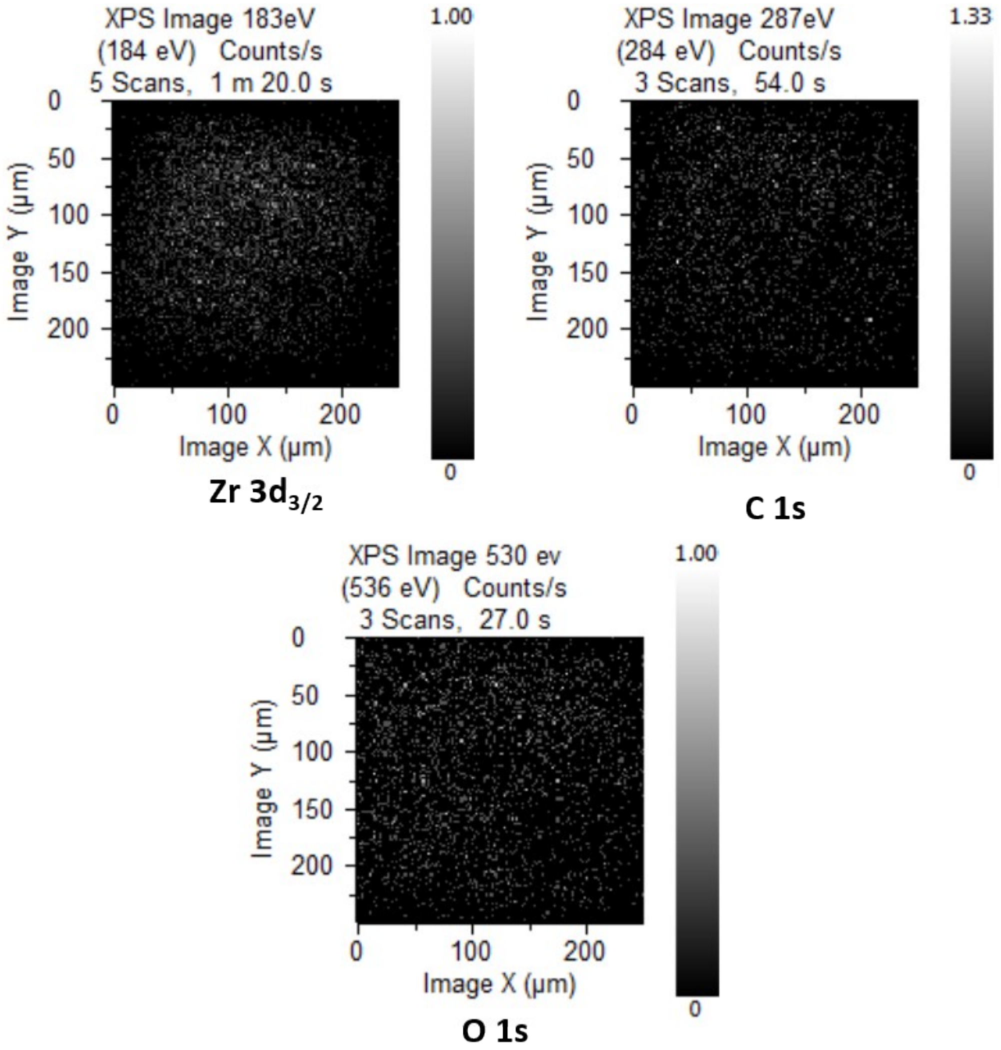

Figure S2 (c): XPS mapping images of Zr-AC.

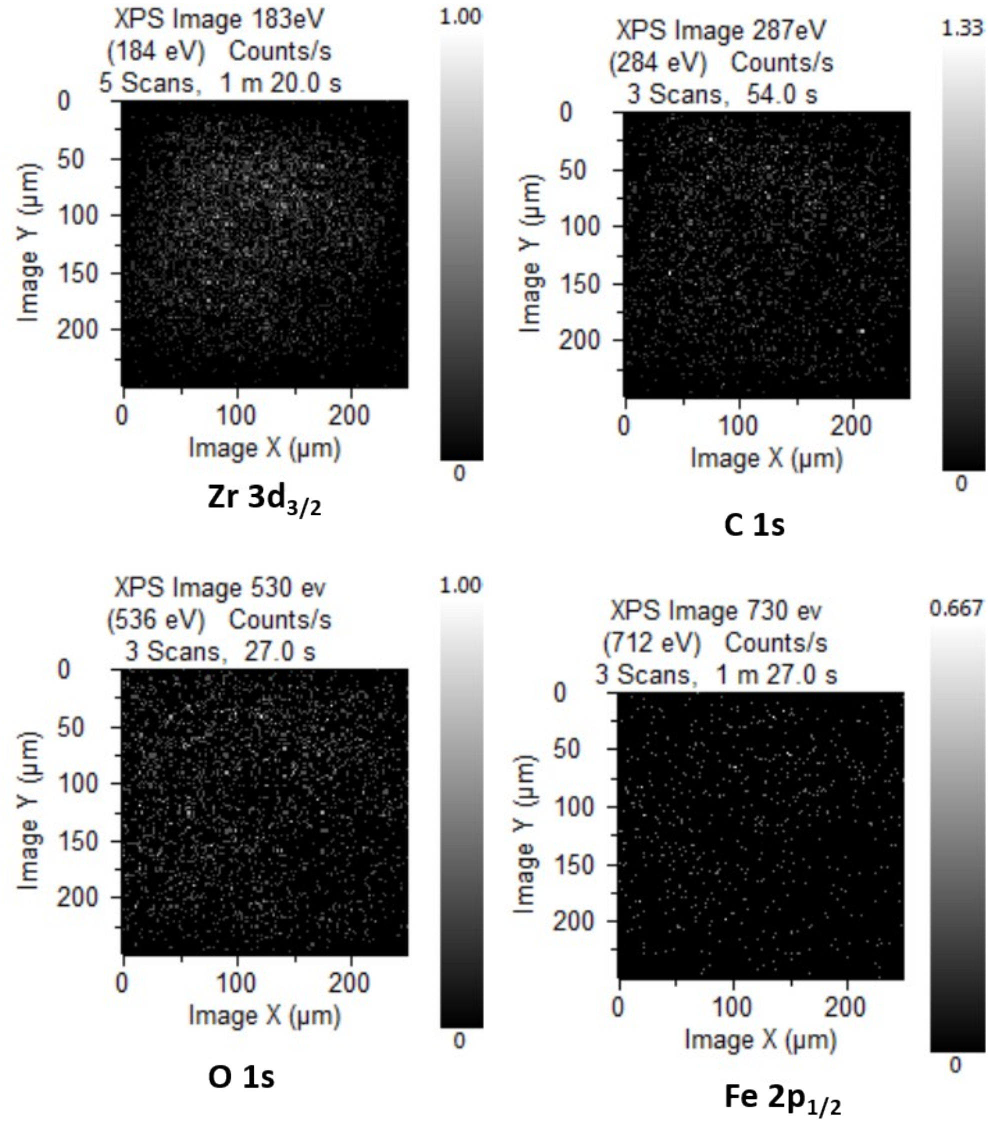

Figure S2 (d): XPS mapping images of FeZr-AC.

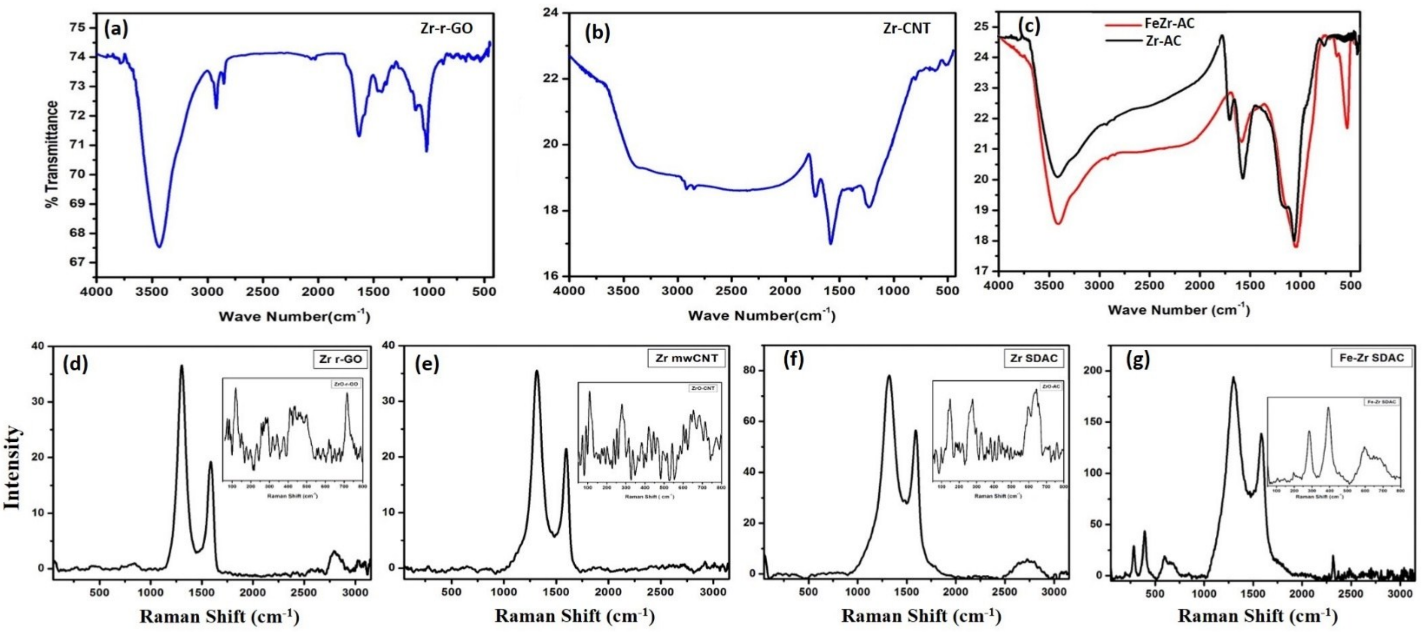

Figure S3: (a), (b), (c) FTIR spectra of ZrO_2 nano particle loaded substrates; **(d), (e), (f), (g)** Raman spectra ZrO_2 nano particle loaded substrates.

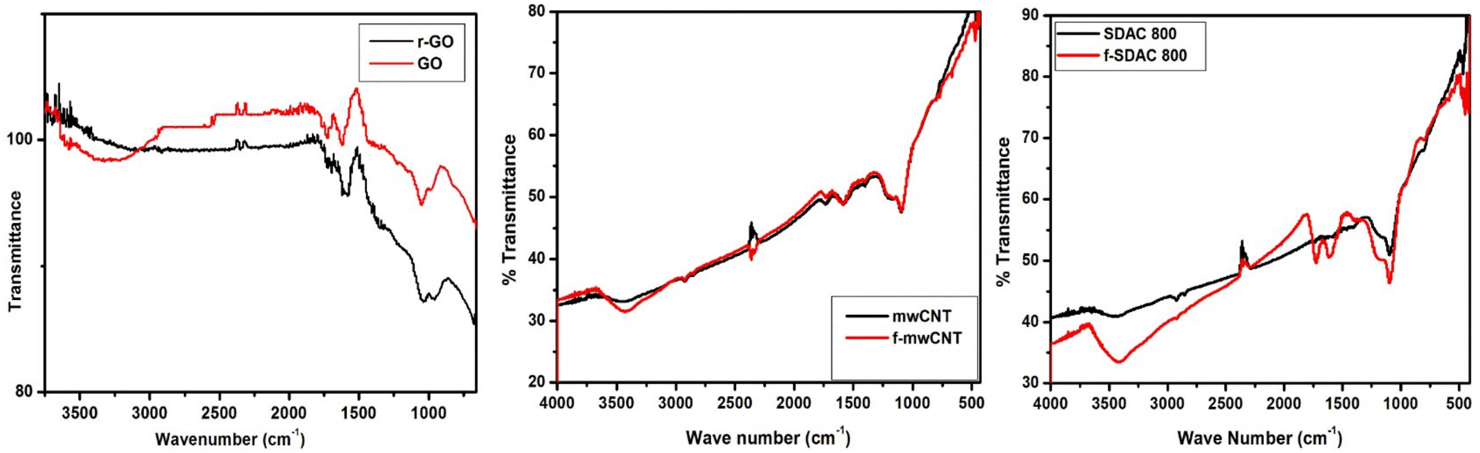

Figure S4: FTIR spectra of plain substrates.

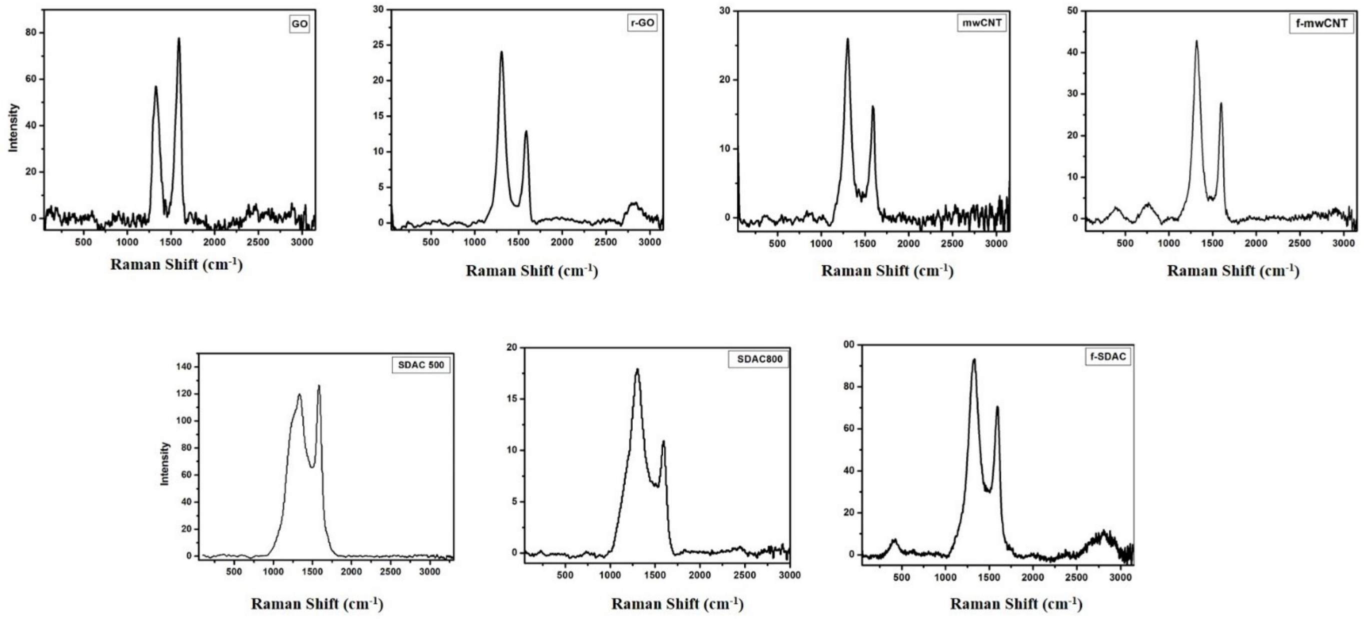

Figure S5: Raman spectra of plain substrates.

Table S1: Raman shifts and Id/Ig of the samples:

Sample	D band (cm $^{-1}$)	G band (cm $^{-1}$)	I_d/I_g
GO	1324	1588	0.734236
r-GO	1302	1586	1.794553
Zr-r-GO	1304	1588	1.875715
mwCNT	1304	1588	1.607054
f-mwCNTs	1315	1597	1.526901
Zr-CNT	1317	1587	1.649604
SDAC500	1331	1586	0.941014
SDAC800	1306	1592	1.645662
F-SDAC800	1324	1596	1.316441
Zr-AC	1324	1596	1.377046
FeZr-AC	1304	1582	1.394953

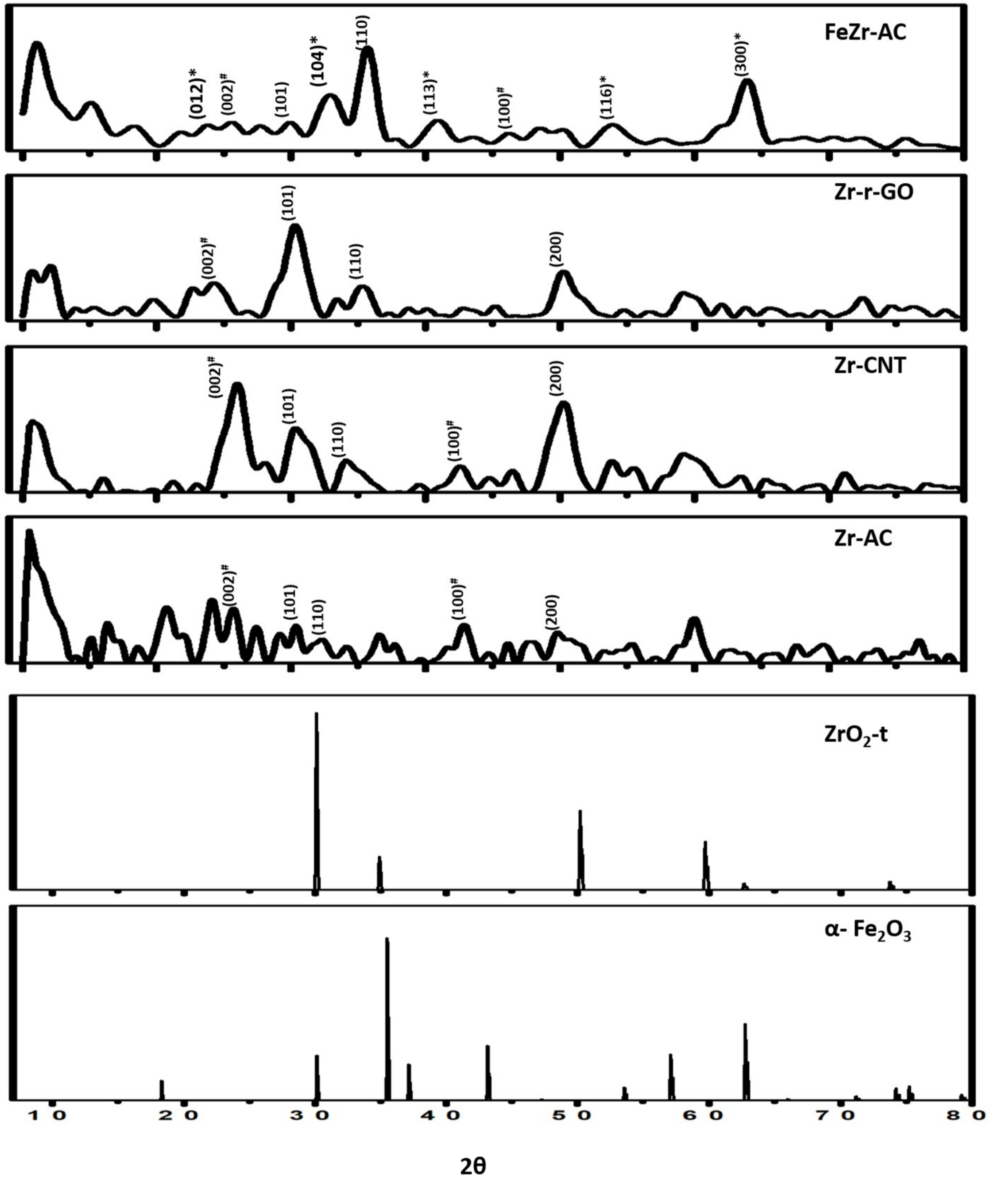

Figure S6: Powder X-ray diffractograms of the Zr-r-GO, Zr-CNT, Zr-AC, and FeZr-AC with reference patterns for tetragonal ZrO_2 and α- Fe_2O_3((*Fe_2O_3, #Carbon structures).

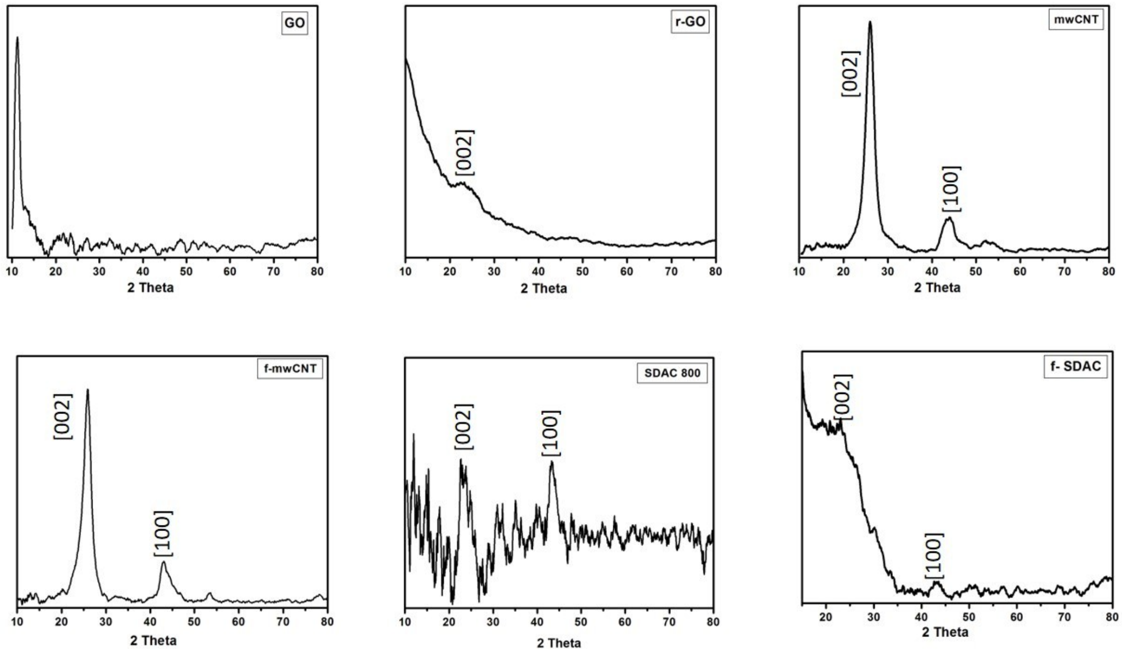

Figure S7: Powder X-ray diffractograms of the carbon materials synthesized.

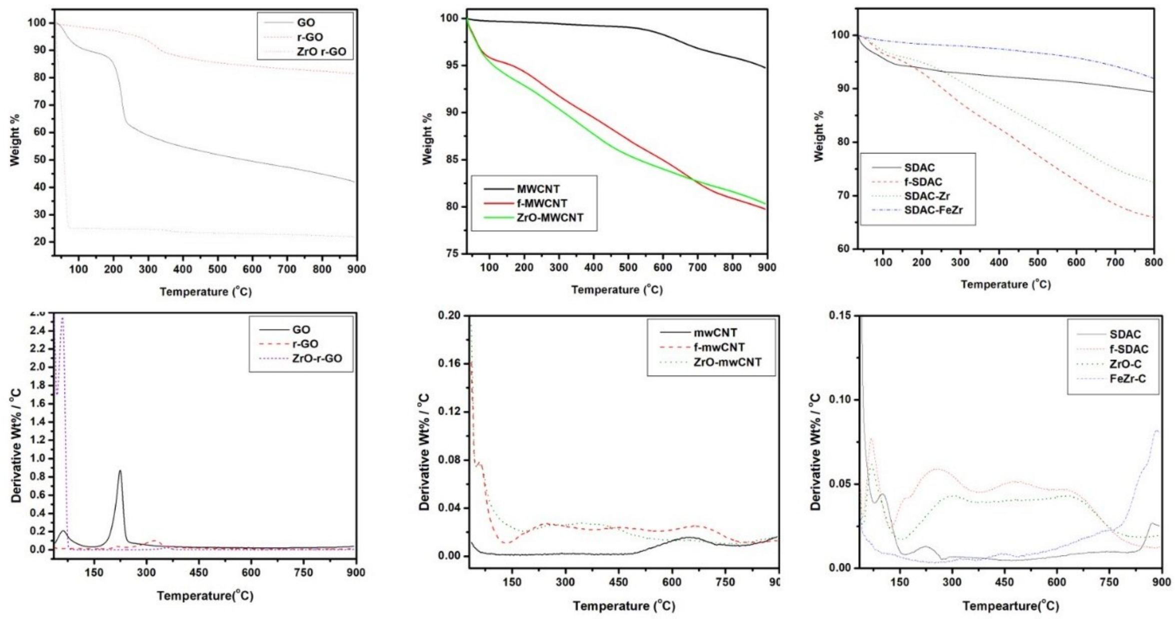

Figure S8: TGA and derivative curves of samples and substrates.

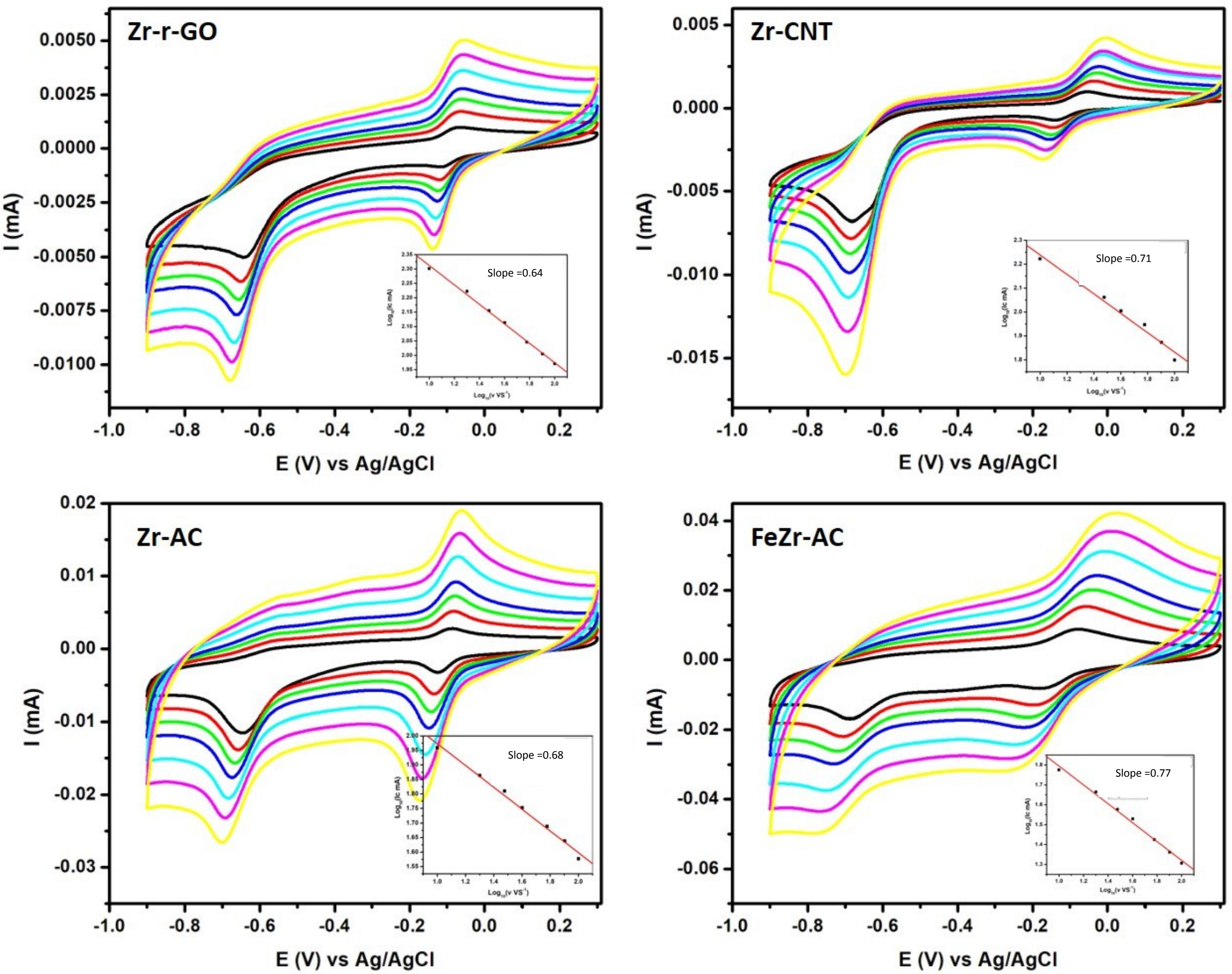

Figure S9: Cyclic voltammograms of the materials Zr-r-GO, Zr-CNT, Zr-AC and FeZr-AC in 15mM PBS (pH 7.2) containing 2.5µgms of MP at a scan rates of 10, 20, 30, 40, 60, 80 and 100 mV/s. Insert: Corresponding logarithmic plots of peak current vs scan rate.

Table S2: Electrochemical Active Surface area (A) and the Electron rate transfer coefficient as calculated from Randles-Sevcik equation and Nicholson equation respectively.

	A (cm^2)	K^o_{obs} (cm s-1)
**Bare GCE**	0.042	2.54×10^{-4}
**Zr-r-GO**	0.35	2.3×10^{-3}
**Zr-CNT**	0.52	2.8×10^{-3}
**Zr-AC**	0.69	2.9×10^{-3}
**FeZr-AC**	0.89	4.1×10^{-3}

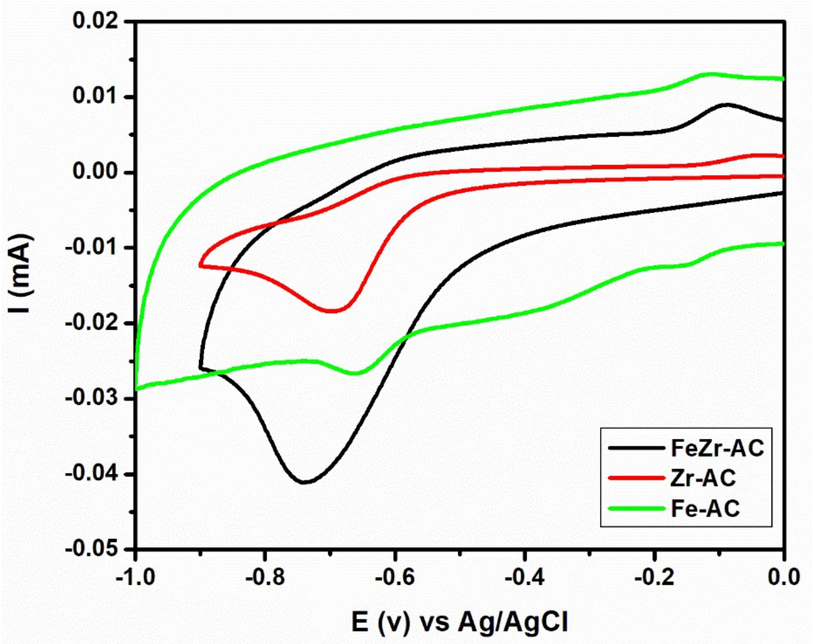

Figure S10: Cyclic voltammograms of the materials Zr-AC, FeZr-AC and Fe-AC in 15mM PBS (pH 7.2) containing 2.5µgms of MP at a scan rate 50 mV/s.

Table S3: Results obtained for real samples analysis using spiked sewage water using the electrochemical method verified against GC-MS

	Zr-r-GO		Zr-CNT		Zr-AC		FeZr-AC	
Added MP (μg. ml^{-1})	0.6	9.0	0.6	9.0	0.6	9.0	0.6	9.0
Recovery (%) (DPV)	99.2	100.3	100.6	100.4	98.9	99.6	100.8	100.3
Recovery (%) (GC-MS)	100.2	99.9	100.2	99.9	100.2	99.9	100.2	99.9

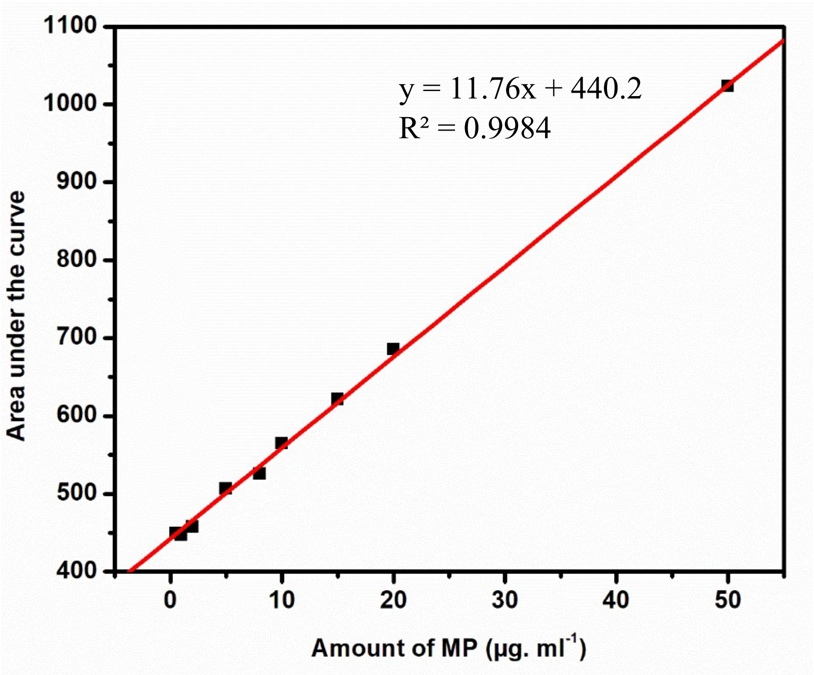

Figure S11: Calibration plot obtained from GC-MS for the analyte MP for different concentrations.

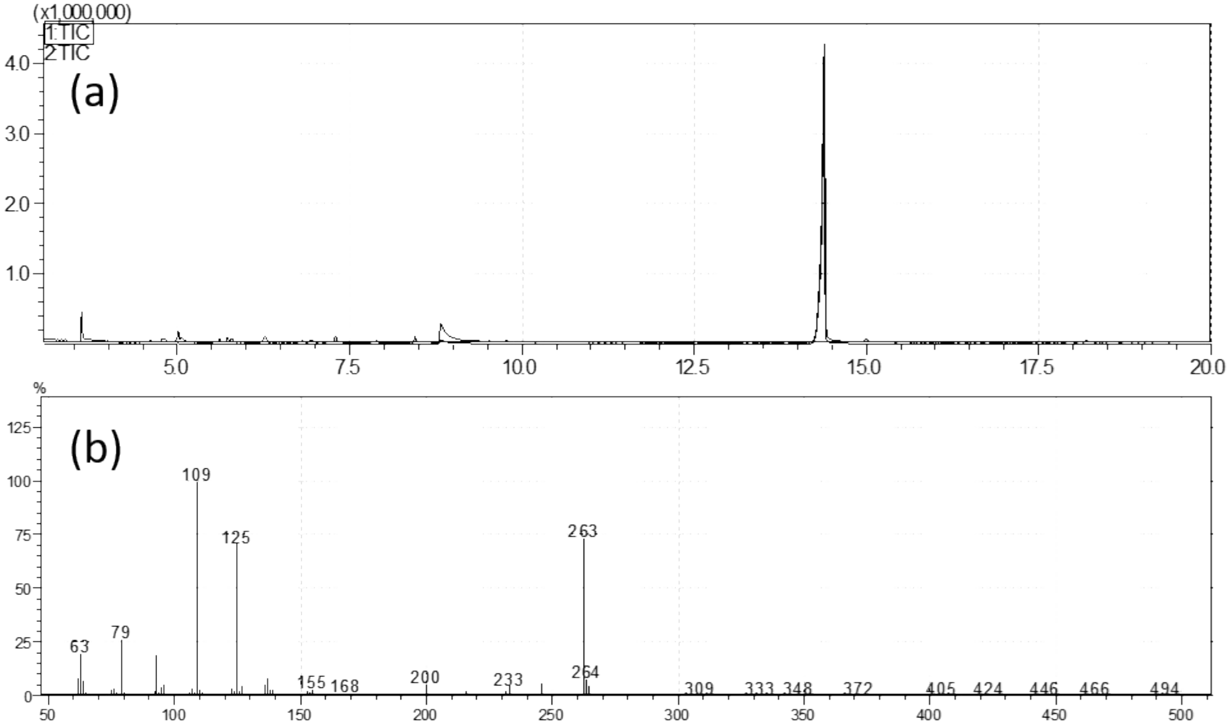

Figure S12: (a) Chromatogram of the analyte MP obtained using GC and (b) corresponding mass spectrum of MP.

Method used:

Column	Schimadzu SH-Rxi-5Sil MS,<br>30 meter x 0.25 mm ID, 0.25 µm.
Injection Temperature	250° C
Transfer Temperature	230° C
Source temperature	200° C
Temperature Program	70° C to 150° C at a rate of 25° C, 150° C. min^{-1} to 200° C at a rate of 5° C. min^{-1}, and 200° C to 280° C at a rate of 8° C. min^{-1} and hold for 2 min.
Injection type	Split (Split ratio is 90:10)
Injection volume	5 µl
Carrier Gas	Helium

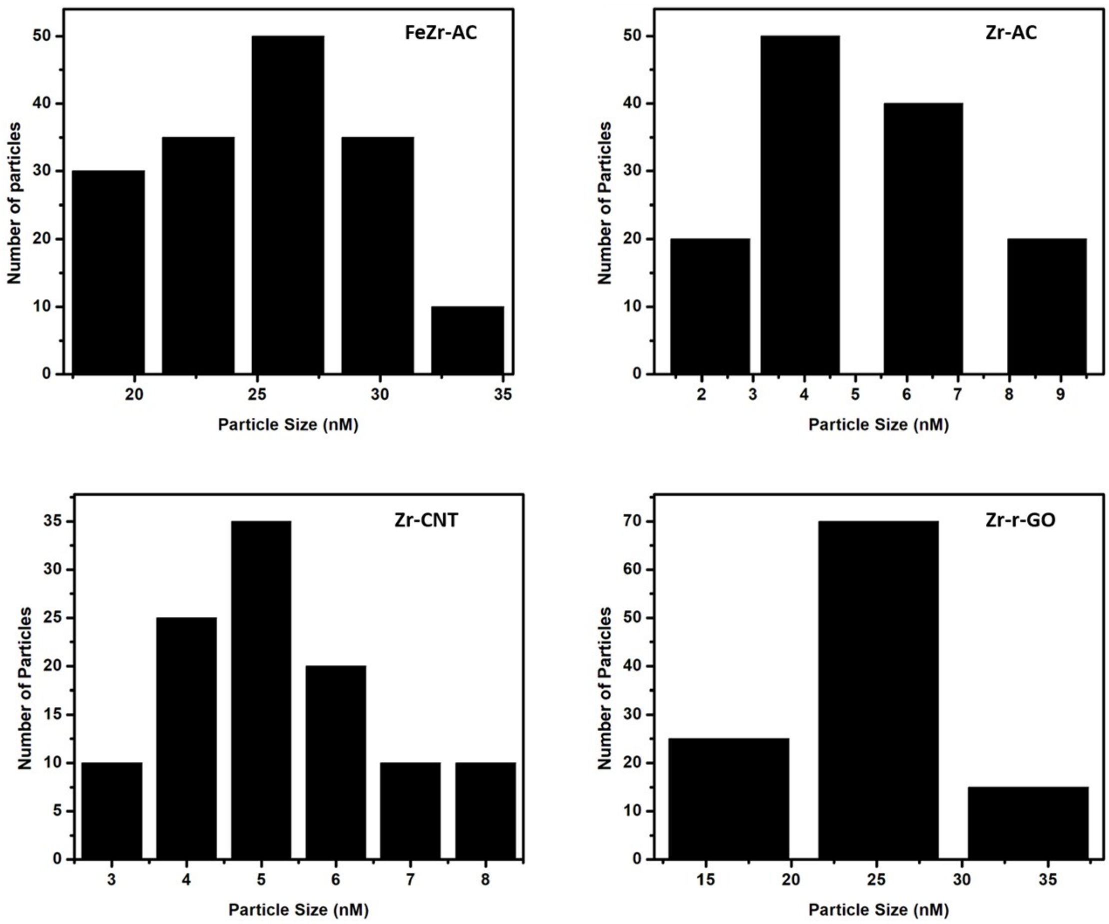

Fig S 13: Histogram showing the particle size distribution in the composites.
